# Ergodicity breaking on the neuronal surface emerges from random switching between diffusive states

**DOI:** 10.1038/s41598-017-05911-y

**Published:** 2017-07-14

**Authors:** Aleksander Weron, Krzysztof Burnecki, Elizabeth J. Akin, Laura Solé, Michał Balcerek, Michael M. Tamkun, Diego Krapf

**Affiliations:** 1 Faculty of Pure and Applied Mathematics, Hugo Steinhaus Center, Wroclaw University of Science and Technology, Wyspianskiego 27, 50-370 Wroclaw, Poland; 2 Department of Biomedical Sciences, Colorado State University, Fort Collins, CO 80523 USA; 3 Department of Biochemistry and Molecular Biology, Colorado State University, Fort Collins, CO 80523 USA; 4 Department of Electrical and Computer Engineering, Colorado State University, Fort Collins, CO 80523 USA; 5 School of Biomedical Engineering, Colorado State University, Fort Collins, CO 80523 USA

## Abstract

Stochastic motion on the surface of living cells is critical to promote molecular encounters that are necessary for multiple cellular processes. Often the complexity of the cell membranes leads to anomalous diffusion, which under certain conditions it is accompanied by non-ergodic dynamics. Here, we unravel two manifestations of ergodicity breaking in the dynamics of membrane proteins in the somatic surface of hippocampal neurons. Three different tagged molecules are studied on the surface of the soma: the voltage-gated potassium and sodium channels Kv1.4 and Nav1.6 and the glycoprotein CD4. In these three molecules ergodicity breaking is unveiled by the confidence interval of the mean square displacement and by the dynamical functional estimator. Ergodicity breaking is found to take place due to transient confinement effects since the molecules alternate between free diffusion and confined motion.

## Introduction

The stochastic motion of molecules in living cells is essential to maintain a myriad of physiological processes. Nevertheless, while diffusion naturally mixes cell components, the cellular environment must be organized in order to maintain a living state. This process is typically fulfilled by actively bringing the system out of thermodynamic equilibrium. In the plasma membrane, different organization levels are observed including the compartmentalization and segregation into functional domains^1–3^, the aggregation into nanoclusters^4, 5^, and the immobilization of macromomecular complexes^6, 7^. Given the multiple biological roles that involve membrane diffusion, its quantitative investigation is highly relevant to cell biology. In general, the organization of the plasma membrane is based on intricate interactions involving the cytoskeleton, membrane proteins, and lipids. During the last decade, our understanding of membrane dynamics and interactions has rapidly evolved due, in a significant fraction, to advances in single-particle localization and tracking^8, 9^. Single-particle tracking (SPT) allows the direct investigation of temporal maturation and spatial heterogeneities. The most typical characterization of individual trajectories is based on the time-averaged (TA) mean square displacement (MSD)

1 $$\overline{{\delta }^{2}({\rm{\Delta }})}=\frac{1}{T-{\rm{\Delta }}}{\int }_{0}^{T-{\rm{\Delta }}}{[{\bf{r}}(\tau +{\rm{\Delta }})-{\bf{r}}(\tau )]}^{2}{\rm{d}}\tau ,$$

where *T* is the experimental time, Δ the lag time, and **r**(*t*) the particle position at time *t*. Note that throughout the manuscript, we employ overlines to indicate time averages and brackets 〈…〉 to indicate averages over an ensemble of particles.

Brownian motion is characterized by a TA MSD that scales linearly in lag time. However, SPT in live cells often reveals complexities accompanied by deviations from normal diffusion^10–12^. In particular the TA MSD can show a non-linear scaling

2 $$\overline{{\delta }^{2}({\rm{\Delta }})}={K}_{\alpha }{{\rm{\Delta }}}^{\alpha },$$

where *K*_*α*_ is the generalized diffusion coefficient and, for subdiffusive processes, 0 < *α* < 1. Besides a non-linear TA MSD, other striking anomalies that can arise in biomolecule dynamics involve ergodicity breaking and aging^13^. We refer to the ergodic property as the equivalence of time and ensemble averages, one of the cornerstones of statistical mechanics.

In living cells, ergodicity breaking has been revealed in the motion of molecules both in the cytoplasm and on the cell surface. In the cytoplasm, the motion of lipid granules in fission yeast cells displays features of weak ergodicity breaking associated with a continuous time random walk (CTRW) subdiffusion with a truncated power-law waiting time distribution^14^. The motion of insulin granules in MIN6 insulinoma cells also exhibits fingerprints of a heavy-tailed CTRW likely due to the random waiting times between binding and unbinding of the granules to microtubules^15^. In the plasma membrane of human embryonic kidney (HEK) cells, ion channels exhibit non-ergodic behaviour because they bind to clathrin-coated pits with a heavy-tailed distribution of immobilization times^7, 16^. The DC-SIGN receptor motion shows ergodicity breaking on the surface of CHO cells caused by heterogeneous dynamics with frequent changes of diffusivity^17^. Furthermore, ergodicity breaking caused by bulk-mediated diffusion was also observed in reconstituted lipid bilayers^18^.

Frequently, when dealing with biological data, only a few trajectories of considerable length are available. If the ergodic hypothesis holds, it is possible to analyse individual sufficiently long trajectories instead of attempting to acquire a large number of measurements, which may not be feasible. In such cases it is critical to assess ergodicity so that temporal and ensemble averages can be interchanged. Classical approaches to demonstrate ergodicity rely on direct comparison of time and ensemble averages or the characterization of the ergodicity breaking parameter, which quantifies the fluctuations of the TA MSDs^19^. However, these tests require numerous realizations of the process. To address such a situation, ergodicity can be tested in a single trajectory employing the concept of dynamical functionals^20–22^.

Here we study ergodicity breaking in the dynamics of membrane proteins on the somatic surface of hippocampal neurons. Neurons are particularly complex cells that require high spatio-temporal regulation and compartmentalization of the plasma membrane in order to properly detect, integrate, and transduce nerve signals. However, the organization of the neuronal surface lacks a thorough understanding. We express three different tagged molecules that traffic to the surface of the soma: the voltage-gated potassium and sodium channels Kv1.4 and Nav1.6, and the glycoprotein CD4. These molecules are found to transiently aggregate into nanoclusters. Interestingly, transient confinement in nanoscale domains causes ergodicity breaking, which is manifested in two different ways. First, significant differences are observed between time- and ensemble-averaged MSD. Second, a dynamical functional test unmasks ergodicity breaking at the individual trajectory level.

## Results

**Figure 1:**
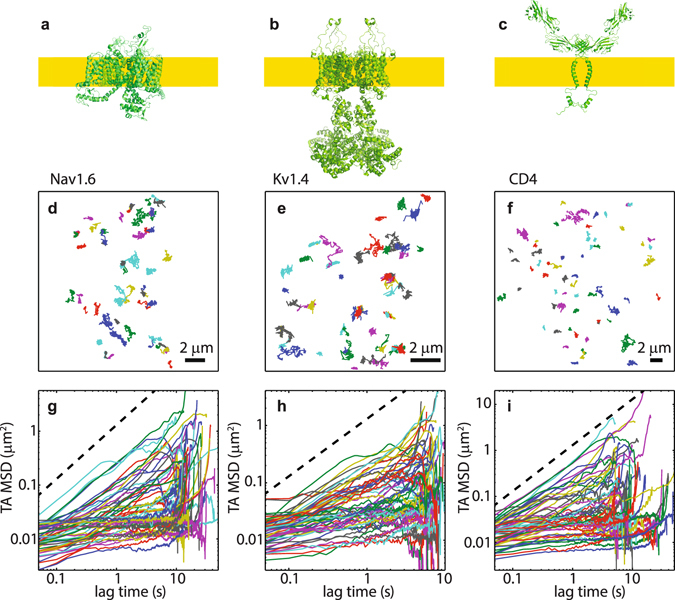
Single-molecule trajectories and their TA MSD. (**a**) Structure of cockroach NavPaS protein (PDB accession code 5X0 M). This is the only solved structure for any eukaryotic Nav channel. (**b**) Structure of Kv1.2 assembled with the beta2 subunit (code 3LUT). (**c**) Structure of CD4 dimer (assembled from codes 1WIO and 2KLU). Structures were produced using PyMOL. The yellow bands in panels a–c represent the plasma membrane with the intracellular protein domains being below the membrane. (**d**–**f**) Trajectories in a representative cells obtained by single-particle tracking. (**g**–**i**) TA MSDs of the trajectories in panels (**d**–**f**). The dashed lines are guides to linear behaviour.

Nav1.6 and Kv1.4 were tagged with an extracellular CF640R fluorophore via biotin-streptavidin. CD4 receptors were tagged with a CF640R-conjugated antibody. Known crystal structures similar to the three molecules studied in this work are shown in Fig. 1a–c as ribbon representations in order to highlight the main features of these molecules and emphasize the differences between them. Figure 1a shows the only solved structure for a eukaryotic Nav channel, the NavPaS protein from cockroach. Note that NavPaS lacks more than 400 cytoplasmic amino acids as compared to the Nav1.6 channel studied in the present work. Thus, the Nav1.6 intracellular domain is larger than the structure pictured in Fig. 1a. No high resolution structure exists for the Kv1.4 ion channel so we show the structure of Kv1.2 complexed with the beta2 subunit in Fig. 1b. The intracellular mass of 2,360 amino acids illustrated in Fig. 1b likely extends farther into the cytoplasm relative to Kv1.4 since Kv1.4 contains only 1,488 cytoplasmic amino acids. Both the Nav and Kv channels contain 24 membrane spanning alpha helices. The CD4 dimer shown in Fig. 1c is the same construct that we are expressing. Surface-labelled molecules are imaged by total internal reflection fluorescence^23^ and tracked using an automated algorithm^24^. We obtained respectively 386, 694, and 1528, Nav1.6, Kv1.4 and CD4 trajectories longer than 100 frames (a statistical analysis of trajectory lengths is presented in Supplementary Fig. S1).

Figure 1d–f show representative trajectories of Nav1.6, Kv1.4, and CD4 proteins, where different trajectories are shown with various colours matched to the TA MSD in Fig. 1g–i. The trajectories depicted in Fig. 1 suggest periods of confinement within nanoscale domains. Previously we have reported that surface Nav1.6 channels in the soma of hippocampal neurons form nanoclusters with a mean radius of 115 nm^23^. Although these clusters are stable for longer than 30 minutes, individual Nav1.6 molecules are seen to associate with and dissociate from these domains at much faster rates. Here we find that nanoscale confining domains also emerge for Kv1.4 and CD4. Marked heterogeneities are observed in the TA MSDs, which can be attributed to molecules with different degrees of confinement and different diffusivities. Most trajectories are classified as subdiffusive, i.e., they display a sublinear TA MSD as in Eq. (2) with *α* < 1.

### Ion channels in the soma of hippocampal neurons exhibit intermittent diffusive behaviour

Qualitative observations indicate that surface molecules in the somatic surface are subjected to transient confinement. Thus we employ an automated algorithm to detect changes in the particle diffusive behaviour. To segment trajectories, we characterize local diffusion by means of a sliding-window TA MSD. It is possible to classify the state according to different parameters such as maximal excursion length^25^, diffusion coefficient^17^, or anomalous exponent *α*. All these metrics provide useful information. We chose to identify the diffusive state via the anomalous exponent because it involves a classification according to a non-dimensional parameter.

**Figure 2:**
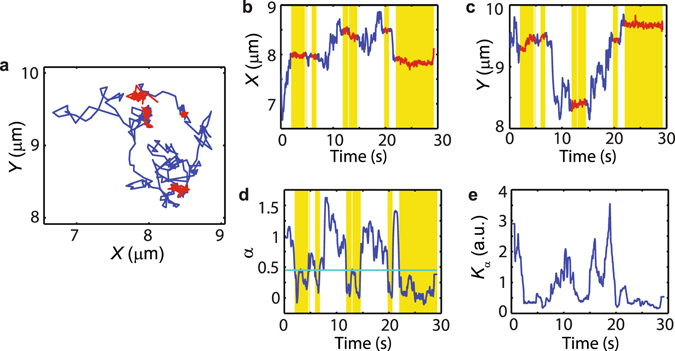
Trajectories are found to alternate between confined and free states. (**a**) Example of Nav1.6 trajectory. The trajectory is coloured according to being in the free or confined state. This trace consists of 600 data points. (**b**,**c**) Time series of the trajectory along *X* and *Y* directions. (**d**) Time series of *α* according to Eq. (2) using a sliding window TA MSD. A threshold *α*_*th*_ = 0.45 as found using a k-means algorithm is employed for discrimination between states. (**e**) Time series of the generalized diffusion coefficient *K*_*α*_ obtained using a sliding window TA MSD.

The classification of the diffusive state according to the anomalous exponent is exemplified in Fig. 2. Figure 2a shows a Nav1.6 trajectory and Fig. 2b,c show the *X* and *Y* positions as a function of time, where the periods in a confined state, as found by a thresholding algorithm, are shaded and marked in red. The local anomalous exponent *α*(*t*) and general diffusion coefficient *K*_*α*_(*t*) are shown in Fig. 2d,e, which are found from a linear regression of log(MSD) vs. log(time) on the first eight time points. The local TA MSD is computed with a sliding window of 23 data points. The anomalous exponent *α*(*t*) exhibits stretches close to one and stretches of low values. When a molecule undergoes confined motion, the TA MSD is bounded by the confining domain and thus it saturates. Thus we foresee that periods of confined motion display a local TA MSD with an anomalous exponent close to zero. In order to find the optimal threshold to resolve low and high *α* values (confined and free states, respectively), we employ the k-means method, a classical classification algorithm^26^. The Nav1.6 data yields a threshold *α*_*th*_ = 0.45.

Thresholding the sliding TA MSD according to the anomalous exponent efficiently determines whether the molecules are in a transiently confined or in a free diffusive state. As seen in Fig. 2a–c, proteins on the somatic surface of hippocampal neurons alternate between two different states. While one of these states displays diffusion over large distances, in a second state the molecules are confined within nanoscale domains. In the cases of Nav1.6 and Kv1.4 most trajectories display 5 to 30 switchings between confined and free states but the number of switchings for CD4 is smaller, because the sojourn times are longer (Supplementary Fig. S2).

### Ergodicity breaking in trajectory dynamics

**Figure 3:**
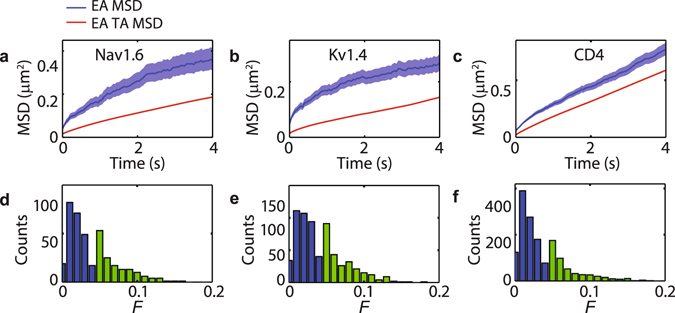
Ergodicity breaking in Nav1.6, Kv1.4, and CD4. (**a**–**c**) EA MSD with the 95% confidence interval vs. EA TA MSD. (**d**–**f**) Dynamical functional test of all trajectories evaluated at *n* = 100 points (5 s). Blue bars correspond to trajectories with *F* < 0.04, whereas the light green bars correspond to *F* ≥ 0.04.

Interestingly, the time traces of the TA MSD $\overline{{\delta }^{2}({\rm{\Delta }})}$ scatter broadly. One possible explanation for this behaviour is rooted in ergodicity breaking^13^. In this scenario, the TA MSD does not converge to the ensemble average. Further, as a consequence of ergodicity breaking the time averages remain random variables in violation of the central limit theorem^27^. In order to test ergodicity, we compare time and ensemble MSDs for each set of molecules. However, given the large scatter of the time averages we study the ensemble average of TA MSD (EA TA MSD), $\langle \overline{{\delta }^{2}({\rm{\Delta }})}\rangle$. Figure 3a–c show both EA MSD and EA TA MSD for Nav1.6, Kv1.4, and CD4. The EA MSD is shown together with the 95% confidence interval^28^. The same figures are shown in logarithmic scales in Supplementary Fig. S3. In all three cases the TA MSD lie outside the EA MSD confidence interval, with the TA MSD being significantly smaller. This difference is a direct indication of ergodicity breaking, with the gap between EA MSD and TA MSD being much larger in Nav1.6 and Kv1.4 than in CD4.

Strong ergodicity breaking arises from the separation of the phase space into uncoupled regions. Thus it can only be assessed in the ensemble of molecules in mutually inaccessible domains. However, when weak ergodicity is broken, the particles are able to explore the whole phase space^29^ and therefore non-ergodic behaviour can be probed at the single-molecule level. This intriguing weakly ergodic behaviour was recently exploited to develop tools to identify non-ergodic dynamics from single-particle trajectories^22^. The inference of ergodicity breaking in single trajectories is based on dynamical functional tests^21, 28^, where the dynamical functional is defined as the time-averaged characteristic function of the normalized increments evaluated for the Fourier mode *ω* = 1. Here, each coordinate of a two-dimensional trajectory (*X*(*n*), *Y*(*n*)) is treated separately, i.e., *X*(*n*) and *Y*(*n*) are restricted to be one-dimensional processes. Given a trajectory *X*(*n*) of *N* points, the normalized increments, i.e., the normalized velocities, are

3 $$V(n)=\frac{X(n+1)-X(n)}{\sqrt{\frac{1}{N-1}{\sum }_{k=1}^{N-1}{[X(k+1)-X(k)]}^{2}}},$$

where we assumed the increments have zero mean. Normalization ensures the method is independent of measurement units. The dynamical functional is then defined as^21^

4 $$E(n)=\langle {e}^{i[V(n)-V\mathrm{(0)}]}\rangle -{|\langle {e}^{iV\mathrm{(0)}}\rangle |}^{2},$$

where 〈…〉 denotes an ensemble average. This functional fully characterizes the ergodic properties of stationary infinitely divisible (SID) processes. A SID process is ergodic if and only if ${n}^{-1}{\sum }_{k=0}^{n-1}E(k)$ goes to 0 as *n* increases^20^. The single-molecule ergodicity estimator is obtained by replacing the ensemble average in Eq. 4 with the time average along the trajectory with *N* + 1 increments *V*(0), *V*(1), …, *V*(*N*),

5 $$\hat{E}(n)=\frac{1}{N-n+1}\sum _{k=0}^{N-n}{e}^{i[V(k+n)-V(k)]}-{|\sum _{k=0}^{N}\frac{{e}^{iV(k)}}{N+1}|}^{2}.$$

The smallness of ${n}^{-1}{\sum }_{k=0}^{n-1}\hat{E}(k)$ for large *n* is the necessary condition for ergodicity, whereas violation of this condition reveals ergodicity breaking. We emphasize that the estimator based on a single trajectory allows to reject, with some degree of certainty, the ergodic hypothesis but it cannot confirm it.

Reliable statistical tests of single-particle trajectories in living cells are desired to identify trajectories for which time averages do not represent the process. Lanoiselée and Grebenkov^22^ introduced the modification to the estimator $\hat{E}(n)$

6 $${\hat{E}}_{\omega }(n,N)=\frac{1}{N-n+1}\sum _{k=0}^{N-n}{e}^{i\omega [X(k+n)-X(k)]}-\frac{1}{N(N+1)}{|\sum _{k=0}^{N}{e}^{i\omega [X(k)-X(0)]}|}^{2}+\frac{1}{N},$$

where the first term can be interpreted as the time-averaged characteristic function of the normalized increments *X*(*k* + *n*) − *X*(*k*) at lag time *n*, and the last term ensures that the estimator is strictly zero for a constant process *X*(*n*) = *X*_0_. The ergodicity estimator generalizes to

7 $${\hat{F}}_{\omega }(n,N)=\frac{1}{n}\sum _{k=1}^{n}{\hat{E}}_{\omega }(k,N),$$

where the summation over *k* is shifted from the original range 0, …, *n* − 1 for convenience. There is a sharp difference between both estimators; we apply Eq. (5) to the short-time increments (or velocities *V*(*k*)) and we apply Eq. (6) to the long-time increments (or positions *X*(*k*)). Let us underline that the high-Fourier modes, with large frequency *ω*, become more important for a finite-length trajectory.

There are three modifications with respect to the original estimator^21^: (i) one can consider Fourier modes beyond *ω* = 1, (ii) the bias is partly removed by subtracting the constant term and changing the normalization, and most importantly, (iii) one can apply the estimators to the long-time increments of a trajectory. Even though the differences between Eqs 5 and 6 are subtle, the application of the improved estimator to the positions of a tracer instead of the short-time increments is a key feature. Following ref. 22, we set *ω* = 2 and for simplicity we denote $F(n)\equiv {\hat{F}}_{2}(n)$. When dealing with SID processes, it is sufficient to consider the estimator defined in Eq. 5 with *ω* = 1. However, the estimator defined in Eq. 6, which depends on *ω*, relaxes the stationarity requirement and thus it allows evaluation of CTRWs. Since the estimator vanishes in the limit that omega increases to infinity for ergodic processes, but remain nonzero for a nonergodic CTRW^22^, the estimation at very large omega might be thought as optimal. However, in practice this strategy is not convenient because of measurement artefacts such as localization errors and blurring. General formulas for any Fourier mode omega are presented in ref. 22 and a thorough analysis is performed in the interval 0 ≤ *ω* ≤ 10. It follows that the choice *ω* = 2 is sufficient for many practical stationary and non-stationary cases.

Ergodicity implies the estimator *F*(*n*) decays to zero, allowing rejection of ergodicity: a large *F*(*n*) confirms ergodicity breaking but smallness of *F*(*n*) does not prove ergodicity. Supplementary Fig. S4a,b show the magnitude of the ergodic estimator |*F*(*n*)|, computed for *X* and *Y* coordinates of the trajectory shown in Fig. 2. We observe that the magnitude related to the *X* coordinate is significantly greater than zero over the whole measurement time, indicating that this trajectory is not ergodic. For comparison, we show in Supplementary Fig. S5 the dynamical functional |*F*(*n*)| for Brownian motion and for fractional Brownian motion with Hurst exponent *H* = 0.35, i.e., anomalous exponent *α* = 0.7^30^. Both these processes are ergodic.

In order to evaluate ergodicity on a single trajectory basis, we compute *F* at *n* = 100 as the maximum of the *X* and *Y* time series estimators, *F* = max(|*F*_*X*_(*n* = 100)|, |*F*_*Y*_(*n* = 100)|). The rationale being that, not to reject ergodicity, the magnitudes of both *F*_*X*_ and *F*_*Y*_ should be small. The value *n* = 100 was chosen in order to allow the comparison of the dynamical function for all trajectories at the same time point (*n* = 100 corresponds to the length of the shortest trajectory). Figure 3d–f show the ergodicity estimator values for Nav1.6, Kv1.4, and CD4 molecules. We employ the concept of *ε*-ergodicity as a statistical test for the ergodic hypothesis. As with other hypothesis tests, an outcome leads to rejection of the hypothesis according to an *ε* significance level, i.e., a probability threshold. In this case the specification of the accuracy *ε* should depend on the experimental noise, the underlying anomalous diffusion process, and the trajectory length. The minimum trajectory length sufficient to identify *ε*-ergodicity breaking was previously evaluated for a large class of anomalous diffusion processes, namely *α*-stable autoregressive fractionally integrated moving average (ARFIMA) processes^30^. In general the minimum required trajectory length depends on the memory parameter, that is the type of random walk. In this work we choose an accuracy *ε* = 0.04, which requires ARFIMA trajectories of length around *n* = 100 as found by Loch-Olszewska *et al*.^30^ We then foresee ergodic trajectories to be characterized by an estimator *F* < *ε* for *n* = 100. However, a large fraction of the trajectories exceeds this value (Fig. 3d–f) indicating *ε*-ergodicity breaking.

### Alternating between diffusive states is responsible for ergodic breaking

**Figure 4:**
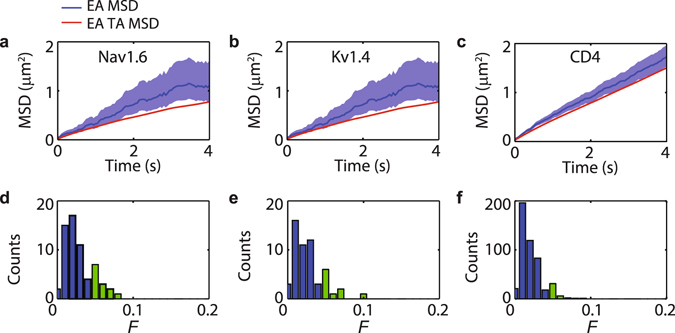
Ergodic behaviour in Nav1.6, Kv1.4, and CD4 of trajectories where the confined states were removed, i.e., analysis of the free motion. (**a**–**c**) EA MSD with the 95% confidence interval vs. EA TA MSD. (**d**–**f**) Dynamical functional test of all trajectories evaluated at *n* = 100 points (5 s). Blue bars correspond to trajectories with *F* < *ε* = 0.04, whereas the light green bars correspond to *F* ≥ 0.04.

We observed that all three studied proteins in the soma of hippocampal neurons exhibit transient confinement in nanodomains and ergodicity breaking. We hypothesize that ergodicity breaking is caused by alternating between confined and free states. Thus we tested this hypothesis by removing the stretches of the trajectories where the molecules exhibit confined behaviour. We then analysed trajectories with an aggregated non-confined behaviour longer than 100 data points (*N* > 100). Figure 4a–c show the EA MSD together with the EA TA MSD of the trajectories without the confined regions. Again, the 95% confidence interval of the EA MSD is shown and the figures are presented in logarithmic scale in Supplementary Fig. S6. While minor differences between EA TA MSD and EA MSD in the free parts of the trajectories can be still observed, these differences are drastically reduced when compared to the raw data (Fig. 3a–c). The differences between the two averages are likely caused by errors in the segmentation of the trajectories, i.e., wrong identifications of free and confined states. Alternatively, a secondary underlying ergodicity-breaking mechanisms could still be functional, e.g., random diffusivities^17, 31^. Nevertheless, our rough confinement identification method indicates that ergodicity breaking in the data is primarily caused by transient confinement.

When the parts of the trajectory with confined behaviour (regions shaded in Fig. 2b,c) are removed and the remaining non-confined parts are stitched together, the dynamical functional test also suggests ergodicity. Supplementary Fig. S4c,d show the ergodic estimator |*F*(*n*)| for *X* and *Y* coordinates of the free state of the trajectory shown in Fig. 2a. For both coordinates we can observe the estimator rapidly converges to zero. Figure 4d–f show the ergodic estimator results for the free part of the trajectories in the three types of membrane molecules. The horizontal axes are the same as in Fig. 3d–f to allow for comparison. The change in behaviour is evident with most of the trajectories now having an ergodic estimator *F* < 0.04 in agreement with ergodic dynamics.

**Figure 5:**
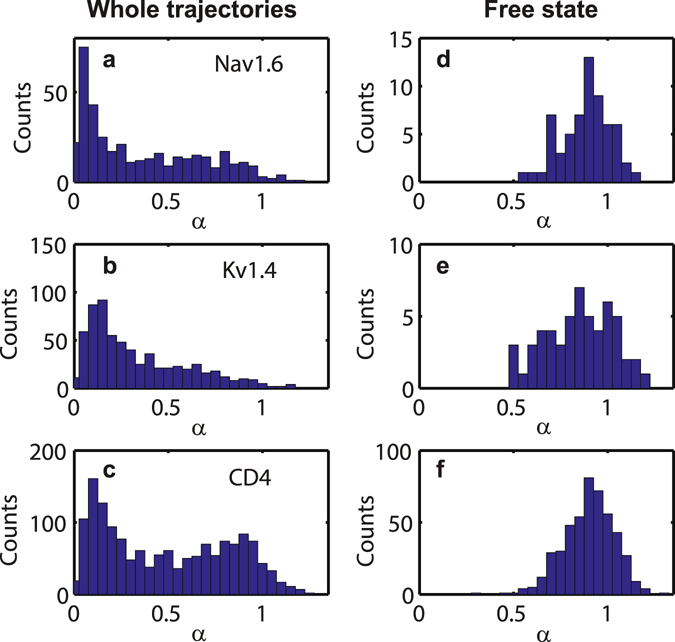
Histograms of TA MSD exponents per trajectory for (**a**) Nav1.6, (**b**) Kv1.4, and (**c**) CD4. The anomalous exponents *α* are computed for whole trajectories. (**d**–**f**) Histograms of TA MSD exponents in Nav1.6, Kv1.4, and CD4, when the confined parts of the trajectories are removed.

The motion of the free (non-confined) state is ergodic but it is still subdiffusive, $\overline{{\delta }^{2}({\rm{\Delta }})}=\langle {x}^{2}(t={\rm{\Delta }})\rangle ={K}_{\alpha }{{\rm{\Delta }}}^{\alpha }$. The anomalous exponents are *α* = 0.90, 0.86, 0.92, respectively for Nav1.6, Kv1.4, and CD4. Statistical analyses of the anomalous diffusion exponents for the whole trajectory and for the free parts are shown in Fig. 5. We observe that the distribution of *α* for the whole trajectories is a mixture of two separated states: free and confined. The TA MSD subdiffusive behaviour of the free parts is characteristic of antipersistent random walks, such as diffusion on a fractal environment^32, 33^ or fractional Brownian motion^34, 35^.

## Discussion

Weak ergodicity breaking has been priorly found in cell membranes due to transient binding^16^ and heterogeneous diffusion landscapes^17^. Non-ergodic behaviour was also observed in the cytoplasm for lipid granules in yeast^14^ and insulin granules in MIN6 cells^15^. To the best of our knowledge, this is the first time ergodicity breaking is found to take place due to transient confinement effects. Given the large intracellular domains of the ion channels Kv1.4 and Nav1.6, one could posit that their confinement is caused by intracellular components. Thus the underlying non-ergodic mechanism of Kv1.4 and Nav1.6 in hippocampal neurons is likely rooted in the cytoplasmic domain as is the case for ergodicity breaking of Kv2.1 channels in HEK cells^16^. Here we report for the first time ergodicity breaking on the neuronal surface. While in HEK cells ergodicity breaking is caused by binding immobilization events, in the complex landscape of the neuronal surface it is caused by transient confinement with a lateral motion of the order of 250 nm.

In terms of renewal theory, ergodicity breaking is attained when the sojourn times in one of the states are power-law distributed, as, e.g., in a continuous time random walk (CTRW)^36^ and in heterogeneous diffusion processes^31, 37^. In the CTRW, a heavy-tail immobilization time distribution leads also to aging^38^. Such behaviour has been experimentally found in systems as diverse as live cells^14–16^, water molecules on cell membrane surfaces^39^, blinking nanocrystals^40–42^, and nanoelectrochemical systems^43^. Similarly non-ergodicity emerges in heterogeneous cell diffusion processes when the distribution of diffusion coefficients has a heavy tail^17^. Nevertheless, the diffusion process described in this work is not renewal and ergodicity breaking could take place without a heavy-tail sojourn-time distribution. Nav1.6 nanoclusters in the soma of hippocampal neurons are stable over long times. Therefore, after escaping from the confining domain, the particle will eventually return to the same domain. Further, the motion of the molecules in the non-confined state is antipersistent as shown by its TA MSD. This subdiffusive behaviour causes the random walk to be more compact than Brownian motion and the return to a domain takes place more readily.

Weak ergodicity breaking lies in the behaviour of individual molecules and not merely at the ensemble level. We have exploited this property and probed ergodicity on a trajectory-by-trajectory basis. It was found that most of the trajectories in the soma surface are non-ergodic. However, when we investigate the non-confined parts of the trajectories, ergodicity is recovered, pinpointing the mechanism behind ergodicity breaking. This behaviour, found for three vastly different membrane proteins emphasizes the neuronal surface complexity, where a classical simple fluid description fails at multiple scales.

## Methods

### Cell culture

Rat hippocampal neurons were cultured and imaged in glass-bottom plates as previously described^23, 44^. Animals were used according to protocols approved by the Institutional Animal Care and Use Committee of Colorado State University (Animal Welfare Assurance Number A3572-01). Dissections were performed after anesthesia with isoflurane followed by decapitation. Hippocampal tissue was dissected from the brains of E18 embryos and neurons were plated on glass-bottom 35-mm dishes with No. 1.5 coverslips (MatTek, Ashland, MA) that had been coated with poly-L-lysine (Sigma-Aldrich, St. Louis, MO) for 1 hr, rinsed with sterile water, then allowed to air dry for 15 min. Neurons were grown in Neurobasal Medium (Gibco/Thermo Fisher Scientific, Waltham, MA) with penicillin/streptomycin antibiotics (Cellgro/Mediatech, Manassas, VA), GlutaMAX (Gibco/Thermo Fisher Scientific), and NeuroCult SM1 Neuronal Supplement (STEMCELL Technologies, Vancouver, BC, Canada). For imaging, the media was replaced by neuronal imaging saline (NIS) consisting of 126 mM NaCl, 4.7 mM KCl, 2.5 mM CaCl_2_, 0.6 mM MgSO_4_, 0.15 mM NaH_2_PO_4_, 0.1 mM ascorbic acid, 8 mM glucose, and 20 mM HEPES (pH 7.4).

### Transfection

Nav1.6 and Kv1.4 constructs were each modified to contain an extracellular biotin acceptor domain (BAD) in an extracellular loop. These constructs (Nav1.6-BAD and Kv1.4-BAD) were previously functionally validated as described^44^. Neuronal transfections were performed after days *in vitro* (DIV) 4–6 in culture using Lipofectamine 2000 (LifeTechnologies, Grand Island, NY) and either Nav1.6-BAD (1 *μ*g), Kv1.4-BAD (1 *μ*g), or CD4 (100 ng), as indicated. For the Nav1.6-BAD and Kv1.4-BAD constructs, pSec-BirA (1 *μ*g) (bacterial biotin ligase) was cotransfected to biotinylate the channel.

### Live-cell surface labelling

Labelling of the surface channel was performed before imaging. Neurons were rinsed with NIS, to remove the Neurobasal media. For experiments with CD4, cells were incubated for 10 min at 37 °C with a monoclonal antibody against CD4 (MABF573, Millipore, Billerica, MA), which we had previously directly conjugated with a CF640R fluorophore (antibody labelling kit Mix-n-stain CF640R, Biotium, Hayward, CA), diluted 1:1000 in NIS. For experiments using the Nav1.6 and Kv1.4 constructs containing the extracellular BAD, cells were incubated for 10 min with streptavidin-conjugated CF640R (Biotium, Hayward, CA) diluted 1:1000 in NIS. Both streptavidin-CF64R and Anti-CD4 labelling was done at 37 °C in the presence of bovine serum albumin (cat. A0281, Sigma, St Louis, MO). Excess label was removed by rinsing with neuronal imaging saline.

### TIRF microscopy

Total internal reflection fluorescence (TIRF) images were acquired using a Nikon Eclipse Ti fluorescence microscope equipped with a Perfect-Focus system, acousto-optic-tunable-filter (AOTF)-controlled 647 nm diode laser, an Andor iXon EMCCD DU-897 camera, and a Plan Apo TIRF 100, NA 1.49 objective. Emission was collected through a filter wheel containing the appropriate bandpass filter. For excitation, an incident angle of 63° was used. Before TIRF imaging, differential interference contrast (DIC) and wide- field fluorescence imaging were used to distinguish transfected neurons from the relatively flat glia. Neurons were readily identified based on the characteristic soma morphology and localization of Nav1.6 to the axon initial segment. All imaging was performed at 37 °C using objective and stage heaters.

### Single-molecule tracking

Rat hippocampal neurons expressing Nav1.6, Kv1.4, or CD4, surface-labelled with CF640R were imaged at 20 frames/s using TIRF microscopy as described above. Images were background subtracted and filtered using a Gaussian kernel with a standard deviation of 0.7 pixels in ImageJ. Tracking of individual fluorophores was then performed in MATLAB using the U-track automated algorithm^24^. Manual inspection confirmed accurate single-molecule detection and tracking.

### Sliding-window MSD

The instantaneous TA MSD at time *t* was found from the detected locations within the trajectories using a sliding-time window averaging method^7^,

8 $${\rm{M}}{\rm{S}}{\rm{D}}({\rm{\Delta }};t)=\frac{1}{n-{\rm{\Delta }}+1}\sum _{k=t}^{t+n-{\rm{\Delta }}}{[{\bf{r}}(k+{\rm{\Delta }})-{\bf{r}}(k)]}^{2},$$

where Δ is the lag time, *n* + 1 is the number of data points in the time series used to calculate the instantaneous TA MSD. The length of the sliding window was set to 23 frames (*n* = 22). Note that the first window contains observations from 1 to *n* + 1, the second from 2 to *n* + 2, etc.

### Algorithm for determination of optimal *α* threshold

For each sliding window we obtain the TA MSD as described above and calculate *α* with Eq. 2. We repeat this procedure for all available trajectories and the new time series *α*(*t*) are classified by using a k-means algorithm. The k-means method provides the minimum value of *α* in each class and the optimal threshold *α*_*th*_ is the minimum of the second class. We have checked the results for values of *n* between 14 and 22 and found the k-means algorithm is robust in terms of window size.

The k-means clustering is a method of vector quantization, originally from signal processing, that is popular for cluster analysis in data mining. The k-means clustering aims to partition *N* observations into *k* clusters in which each observation belongs to the cluster with the nearest mean, serving as a prototype of the cluster. This method yields a partitioning of the data space into Voronoi cells^26^. The k-means algorithm is the standard procedure implemented in various numerical packages, including MATLAB.

## Electronic supplementary material

Supplementary Figures
