# Supplementary material for: Ergodicity breaking on the neuronal surface emerges from random switching between diffusive states
Source: Sci Rep. 2017 Jul 14;7:5404. doi: 10.1038/s41598-017-05911-y (PMC5511290; doi:10.1038/s41598-017-05911-y)
Supplement: Supplementary file 1 — Supplementary Figures [file 41598_2017_5911_MOESM1_ESM.pdf]

Supplemental material for "Ergodicity breaking on the neuronal surface emerges from random switching between diffusive states" by A. Weron, K. Burnecki, E. J. Akin, L. Solé, M. Balcerek, M. M. Tamkun, and D. Krapf

List of Supplementary Figures

1. Histograms of trajectory lengths
2. Histograms of number of switchings between confined and free state
3. Comparison of EA MSD and EA TA MSD
4. Dynamical functional $|F(n)|$ of individual trajectory
5. Dynamical functional test $|F(n)|$ with respect to n for Brownian motion and fractional Brownian motion
6. Comparison of EA MSD and EA TA MSD in the free parts of the trajectories
7. Scatter plots of generalized diffusion coefficient K_α vs. anomalous exponent α

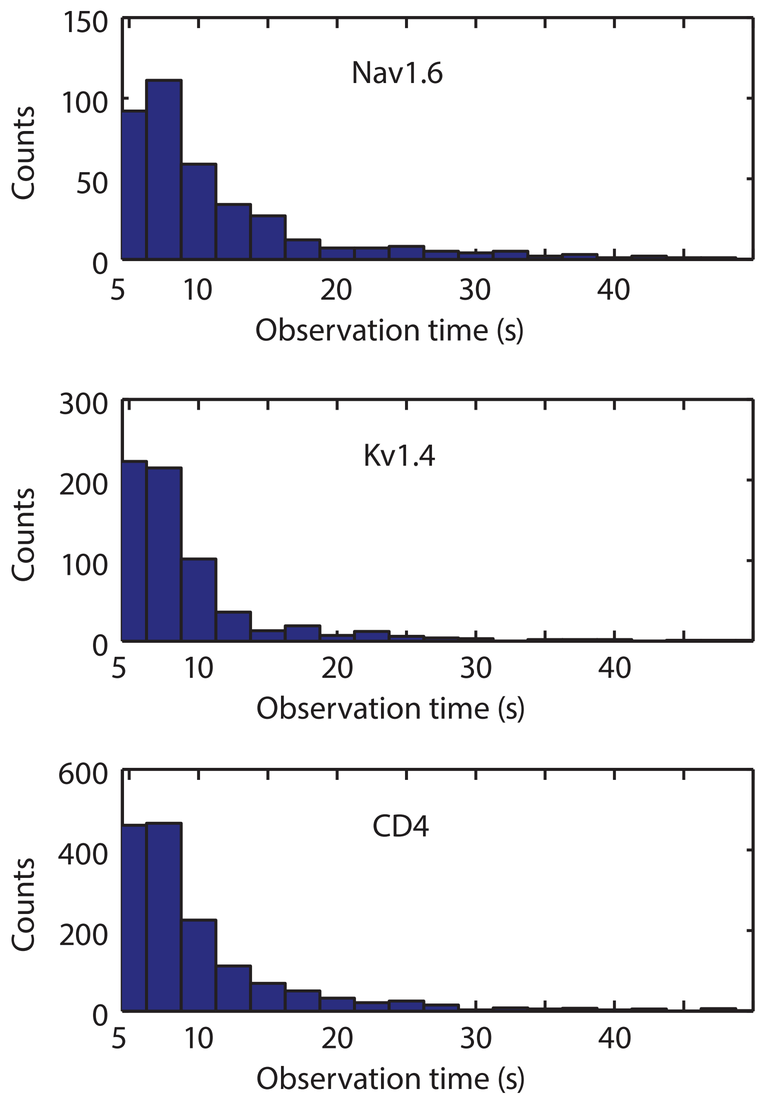

Figure S1: Histograms of trajectory lengths for (top panel) Nav1.6, (middle panel) Kv1.4, and (bottom panel) CD4.

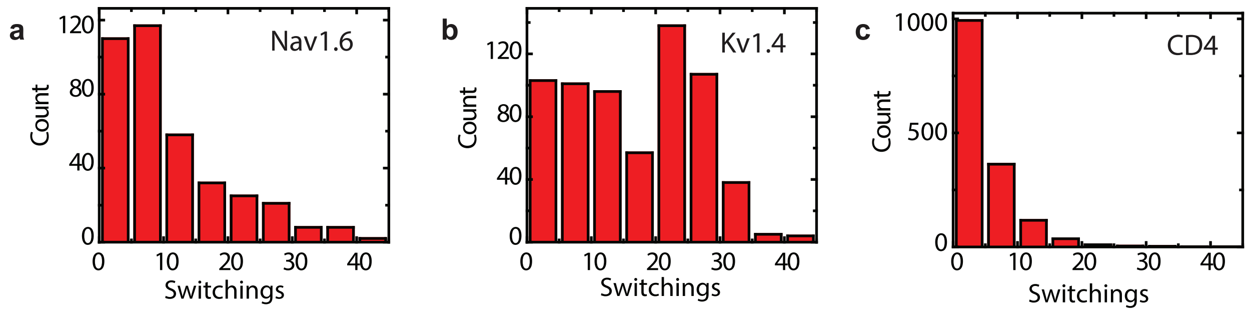

Figure S2: Histograms of number of switchings between confined and free state per trajectory for (a) Nav1.6, (b) Kv1.4, and (c) CD4.

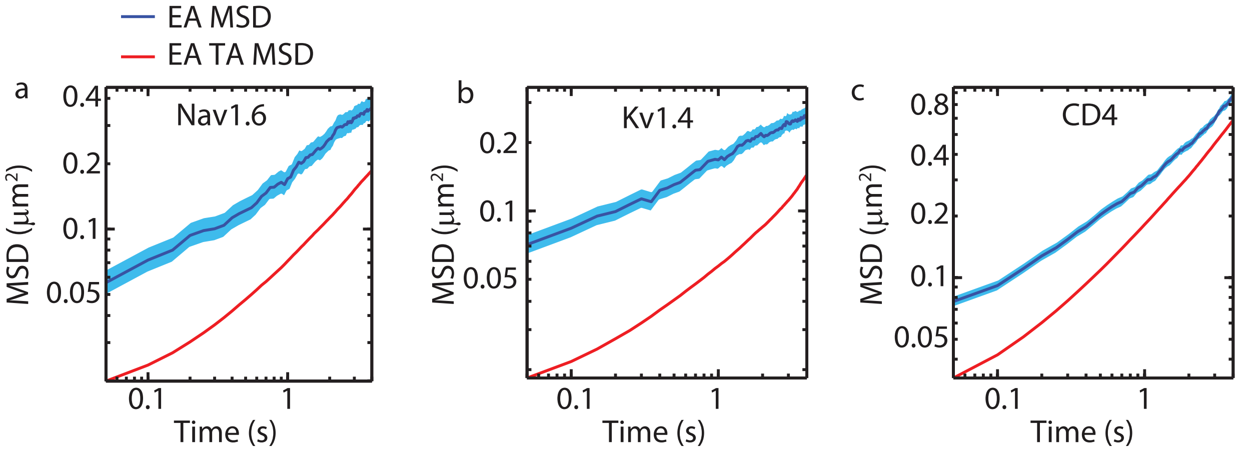

Figure S3: Comparison of EA MSD and EA TA MSD. The EA MSD is shown with the 95% confidence interval as a shaded region. (a) Nav1.6, (b) Kv1.4, and (c) CD4. The plots are the same as the ones presented in Figure 3 in the main text, but here they are shown in logarithmic scales, which allows comparison of the anomalous exponent.

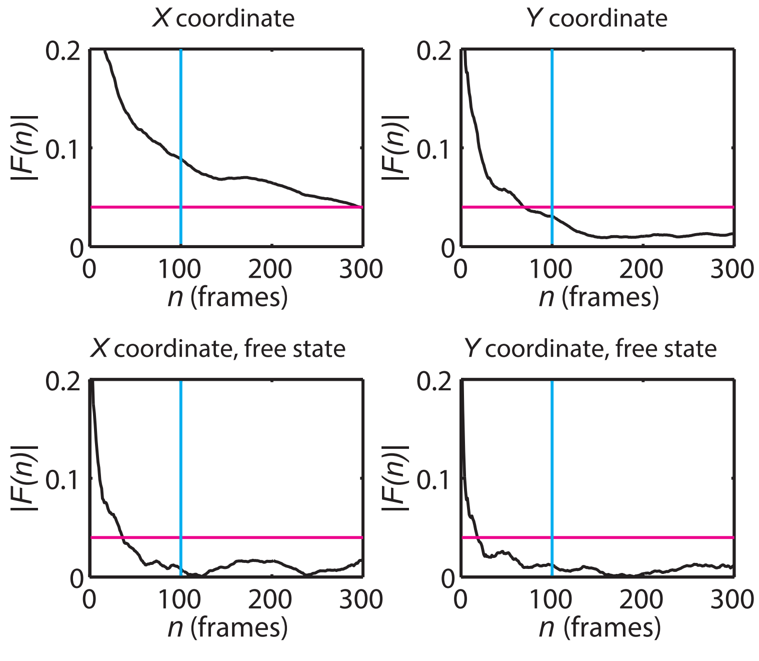

Figure S4: Dynamical functional $|F(n)|$ behavior of the single trajectory shown in Fig. 2a in the main text as a function of observation time n along X (left panel) and Y (right panel) directions. Top panel shows the behavior for the whole trajectories and bottom panel of the free-state parts. The horizontal magenta lines correspond to the $\epsilon = 0.04$-accuracy level and the vertical cyan lines to $n = 100$, as used in the analysis.

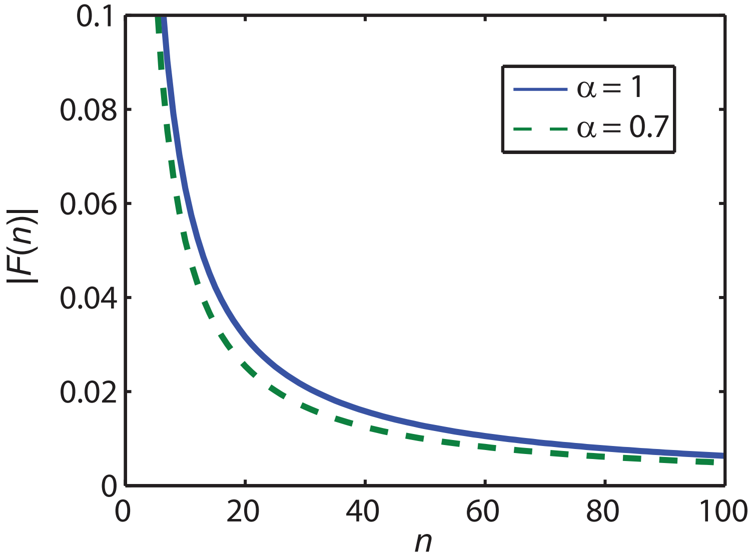

Figure S5: Dynamical functional test $|F(n)|$ with respect to n for two ergodic Gaussian processes, namely Brownian motion ($\alpha = 1$) and fractional Brownian motion with Hurst exponent $H = 0.35$ ($\alpha - 0.7$). The general equation for the dynamical functional test for Gaussian processes are given in Eq. 19 in Ref. 30.

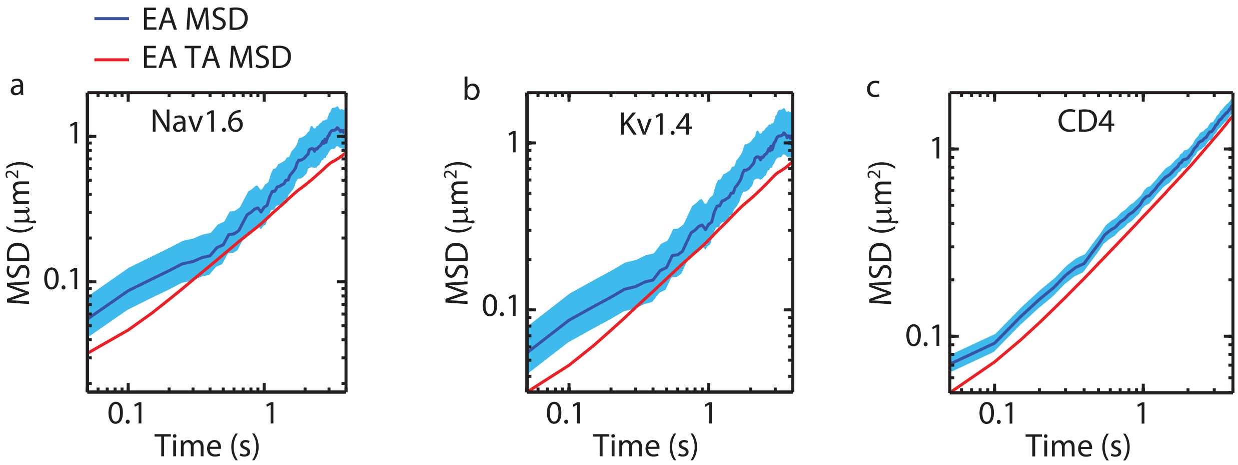

Figure S6: Comparison of EA MSD and EA TA MSD in the free parts of the trajectories. The EA MSD is shown with the 95% confidence interval as a shaded region. (a) Nav1.6, (b) Kv1.4, and (c) CD4. The plots are the same as the ones presented in Figure 4 in the main text, but in logarithmic scales. Comparison with Fig. S3 emphasizes how in the free state the averaged TA MSD approaches the EA MSD while in the whole trajectories the differences are much larger.

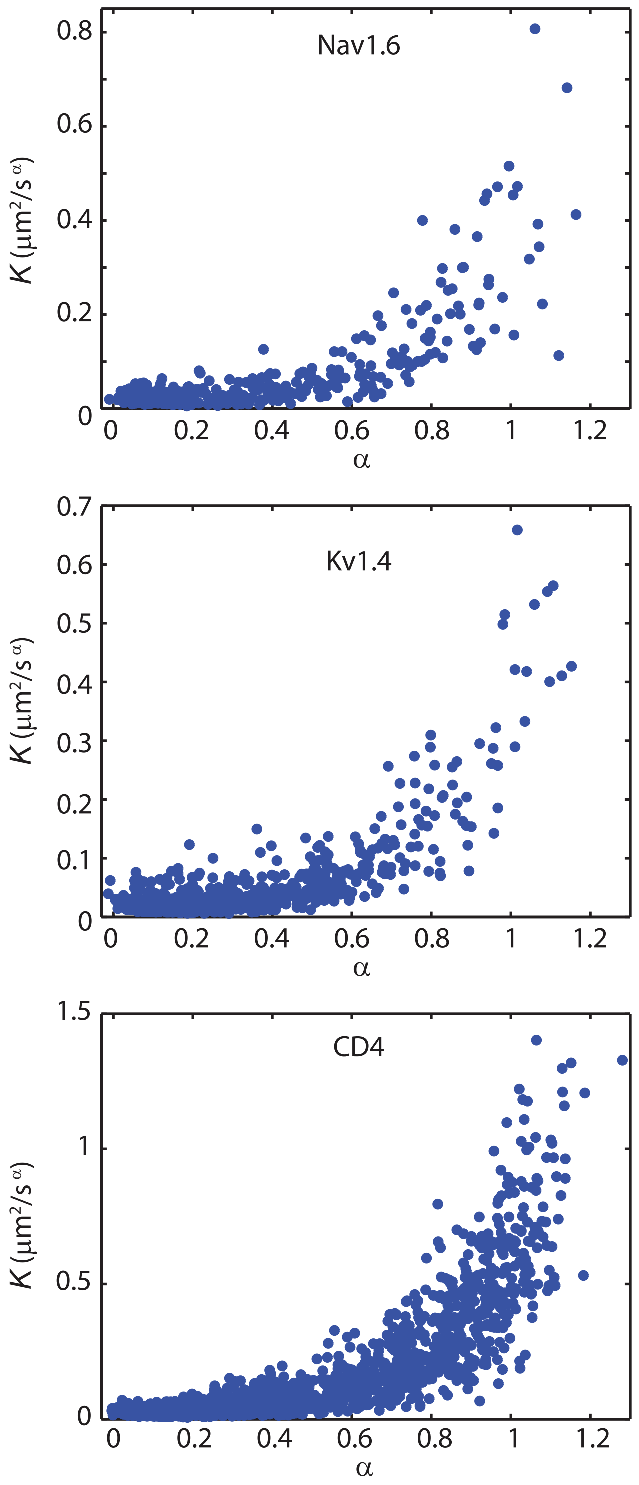

Figure S7: Scatter plots of generalized diffusion coefficient K_α vs. anomalous exponent α computed from the time-averaged MSD $\overline{\delta^2(\Delta)} = K_\alpha \Delta^\alpha$. It is observed that in all three membrane proteins K_α and α are positively correlated.
